# The Complex Network between MYC Oncogene and microRNAs in Gastric Cancer: An Overview

**DOI:** 10.3390/ijms21051782

**Published:** 2020-03-05

**Authors:** Ana Carolina Anauate, Mariana Ferreira Leal, Danielle Queiroz Calcagno, Carolina Oliveira Gigek, Bruno Takao Real Karia, Fernanda Wisnieski, Leonardo Caires dos Santos, Elizabeth Suchi Chen, Rommel Rodríguez Burbano, Marília Arruda Cardoso Smith

**Affiliations:** 1 Disciplina de Genética, Departamento de Morfologia e Genética, Universidade Federal de São Paulo, São Paulo SP 04023-062, Brazil; anauatte@gmail.com (A.C.A.); lealmf@gmail.com (M.F.L.); carolina.gigek@unifesp.br (C.O.G.); brunokaria@gmail.com (B.T.R.K.); f.wisnieski@unifesp.br (F.W.); caires.leonardo@unifesp.br (L.C.d.S.); eschen@unifesp.br (E.S.C.); 2 Disciplina de Nefrologia, Departamento de Medicina, Universidade Federal de São Paulo, São Paulo SP 04023-062, Brazil; 3 Núcleo de Pesquisas em Oncologia, Hospital Universitário João de Barros Barreto, Universidade Federal do Pará, Belém PA 66075-110, Brazil; danicalcagno@gmail.com (D.Q.C.); rommel@ufpa.br (R.R.B.); 4 Departamento de Patologia, Universidade Federal de São Paulo, São Paulo SP 04023-062, Brazil; 5 Disciplina de Gastroenterologia, Departamento de Medicina, Universidade Federal de São Paulo, São Paulo SP 04023-062, Brazil; 6 Laboratório de Citogenética Humana, Instituto de Ciências Biológicas, Universidade Federal do Pará, Belém PA 66075-110, Brazil; 7 Laboratório de Biologia Molecular, Hospital Ophir Loyola, Belém PA 66063-240, Brazil

**Keywords:** gastric cancer, microRNA, 8q24.21, MYC, target therapy

## Abstract

Despite the advancements in cancer treatments, gastric cancer is still one of the leading causes of death worldwide. In this context, it is of great interest to discover new and more effective ways of treating this disease. Accumulated evidences have demonstrated the amplification of 8q24.21 region in gastric tumors. Furthermore, this is the region where the widely known *MYC* oncogene and different microRNAs are located. MYC deregulation is key in tumorigenesis in various types of tissues, once it is associated with cell proliferation, survival, and drug resistance. microRNAs are a class of noncoding RNAs that negatively regulate the protein translation, and which deregulation is related with gastric cancer development. However, little is understood about the interactions between microRNAs and MYC. Here, we overview the MYC role and its relationship with the microRNAs network in gastric cancer aiming to identify potential targets useful to be used in clinic, not only as biomarkers, but also as molecules for development of promising therapies.

## 1. Introduction

Gastric cancer (GC) is one of the most common cancers worldwide, and its incidence is very high mainly in Eastern Asia, Eastern Europe, and South America [1,2]. In 2018, the Global Cancer Observatory (GLOBOCAN) estimated 1,033,701 new cases of stomach neoplasms worldwide, representing 5.7% of all new cases of cancer. Additionally, gastric tumors were expected to be the 3rd cause of cancer related death among males and the 5th among females, evidencing that GC is more likely to be diagnosed in males than in females. Collectively, between men and women, stomach cancer was predicted to correspond to 782,685 deaths and be the 7th most prevalent neoplasm. Moreover, for 2018, globally, the risk of developing stomach cancer was 1.87% in men and 0.79% in women. While the risk of dying because of stomach cancer was 1.36% for males and 0.57% for females [3]. GC is an aggressive disease commonly diagnosed at advanced stages, and surgical resection associated with chemotherapy or chemoradiation is considered the main treatment option for GC [4]. The etiology of GC is multifactorial including environmental factors, such as *Helicobacter pylori* (*H. pylori*) infection [5] and Epstein–Barr virus infection [6], dietary factors [7,8], high consumption of alcohol [9], smoking [10], as well as genetic factors [11], and epigenetic alterations [12,13].

**Figure 1:**
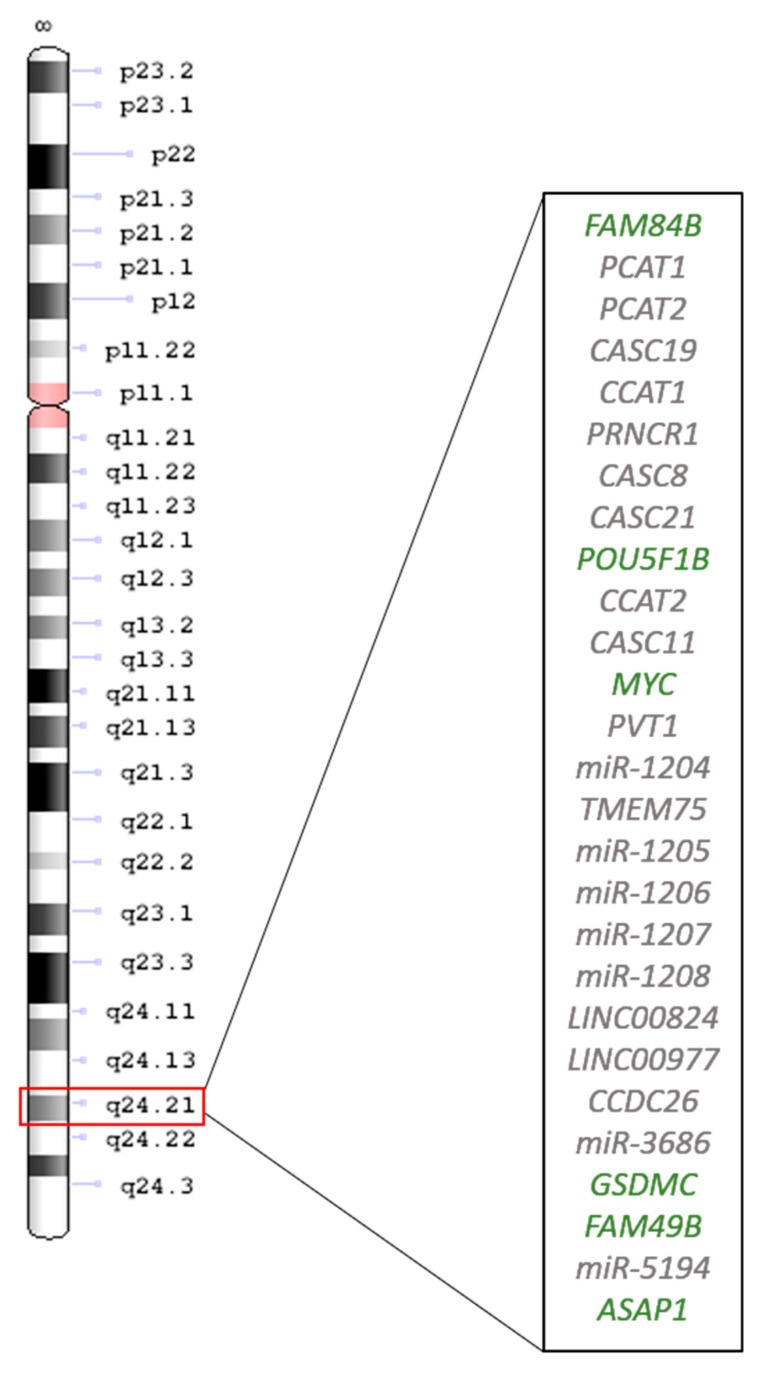
The 8q24.21 genes. The coding genes are shown in green, and the non-coding genes in grey.

Chromosome 8 regions are frequently amplified in GC samples, especially the 8q24.21 region [14,15,16,17]. Among the coding genes present in this region, the most studied is *MYC* (also named *c-MYC*; Figure 1), and the literature shows that its activation can contribute to tumorigenesis [18,19,20]. *MYC* amplification and its upregulated expression have been commonly observed in GC cell lines and GC tissues, and the highest *MYC* levels have been reported in the tumors of patients with local or distant metastasis [14,17,21,22,23,24,25,26,27,28,29]. The key role of MYC in GC etiology was further confirmed in a nonhuman primate model, where both *MYC* expression and copy number were continuously increased during the sequential steps of intestinal-type gastric carcinogenesis [26]. Indeed, *MYC* not only has a key role in gastric carcinogenesis but is also one of the most robust and significant prognostic markers of GC [16]. For this reason, further topics will explore more of this role.

It is worth mentioning other coding and noncoding genes of 8q24.21, shown in Table 1. Among them, the plasmacytoma variant translocation 1 (*PVT1*) oncogene encodes a long noncoding RNA (lncRNA). Compared with *MYC*, *PVT1* is less studied, but it is involved in critical processes in cancer cells, including DNA rearrangements, genetic instability, microRNA (miRNA) encoding, and also interacts with *MYC* itself [30,31,32,33]. Increased *PVT1* expression was shown to induce cell proliferation and migration in GC cell lines, and it was previously associated with higher cell invasion, advanced stages, and poor prognosis in GC patients [34,35,36]. Besides *PVT1*, the *CASC11* noncoding gene is highly expressed in GC tissues and cell lines, and its knockdown inhibits cancer progression [37,38,39,40,41,42].

Some miRNAs (Table 1) were also identified in the 8q24.21 region. miRNA is a molecular class of small noncoding RNA of approximately 22 nucleotides that regulate gene expression through sequence complementarity with the target mRNA. miRNA genes are transcribed into primary miRNA transcripts and subsequently processed by the *RNase III* enzyme *Drosha* inside the nucleus, releasing 60-110-nucleotide pre-miRNA hairpins. The pre-miRNA is then exported into the cytoplasm by *Exportin-5*, where it is cleaved by *Dicer* into ~22-nucleotide double-stranded miRNAs. Finally, miRNAs regulate the expression of their mRNA targets when the multiprotein RNA-induced silencing complex (RISC) is formed [50]. In this process, total complementarity results in the cleavage of the mRNA target strand, while imperfect complementarity leads to repression of the mRNA translation [51]. Thus, unsurprisingly, miRNA deregulation has been described in different diseases, including GC [47,52], and this deregulation may help us elucidate critical pathways involved in carcinogenesis processes and identify potential prognostic or predictive biomarkers [53,54,55,56].

Notably, a variety of miRNAs can also directly or indirectly regulate M*YC* expression [57,58]. Therefore, the complex interaction between *MYC* and miRNAs still needs to be further understood. This review updates and illustrates the oncogenic role of *MYC* in gastric carcinogenesis and its association with *H. pylori* infection, highlighting the network with miRNAs.

## 2. Biological Significance of *MYC*

The *MYC* family is a group of cellular proto-oncogenes with the following three highly related nuclear phosphoproteins: MYC, N-MYC, and L-MYC [59]. MYC has a low expression and has a short half-life in normal cells, and its mRNA level is tightly regulated by both transcriptional and post-transcriptional mechanisms [60]. However, it is overexpressed in several neoplasms. Our group and others have shown *MYC* overexpression in GC samples [17,43,44], including early stages [23,61], and reported MYC protein overexpression [23,24]. Moreover, other studies revealed the importance of the co-amplification of *MYC* and *EGFR* and *FGFR2*, in predicting poor survival of patients undergoing cancer therapy [62]. In tumor cells, *MYC* activation occurs as follows: (1) mutations in signaling pathways proteins upstream from MYC; (2) mutations and single nucleotide polymorphisms in regulatory regions that enhance the stability of these proteins [63] and (3) direct modification of *MYC* gene via gene amplification, mutation, chromosomal translocation and epigenetic modifications [24,63,64,65].

**Figure 2:**
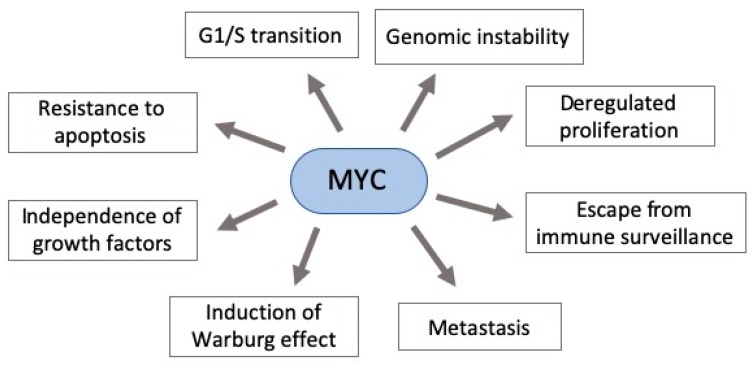
Pleiotropic consequence of MYC deregulation in cancer. MYC overexpression in gastric carcinogenesis affects various components of signaling pathways critical to cancer establishment. Some of these pathways’ phenotypes are shown here.

MYC deregulation plays an important role in neoplastic development by targeting genes involved in critical cellular functions, such as DNA metabolism and dynamics, cell cycle, apoptosis, adhesion, survival, and protein and macromolecular synthesis [60,66,67]. Moreover, it contributes to aerobic metabolism by activating the expression of several genes essential for glycolysis and mitochondrial biogenesis [68]. Additionally, its hyperactivity can allow widespread miRNAs downregulation through the regulation of transcriptional and post-transcriptional mechanisms. Indeed, *MYC* is known as the gene with the highest interaction with downregulated miRNAs [69,70,71]. Taken together, this scenario shows that MYC deregulation (usually overexpression) can have an impact in various cellular functions, contributing to an abnormal cell growth (Figure 2) [68,72].

Infectious agents are extremely important factors on cancer development, accounting for 16% of all new cancer cases per year worldwide [73]. Moreover, liver and gastric tumors in men account for greater than 80% of the infection-related burden cancers [73]. According to the International Agency for Research on Cancer (IARC), 78% of all GC cases are estimated to be associated to chronic *H. pylori* infection, a bacteria classified as a group 1 carcinogen [74]. The virulence of this bacterium is commonly determined by c*agA* and v*acA* genes. The *cagA* gene encodes the secretion complex, capable of introducing the cagA oncoprotein in the gastric epithelial cell, which activates mitogen-activated protein (MAP) kinases. This alteration activates cell proliferation, differentiation, and stress and inflammatory responses and inhibits programed death, leading to a precancerous process [75,76]. Especially in intestinal-type of GC, *H. pylori* cagA has been associated with increased *MYC* expression and nuclear MYC protein [77,78]. In *H. pylori* infected patients with active gastritis, chromosomal aneuploidy and cellular DNA damage were associated with *MYC* expression, leading to a chronic hyperproliferation [79]. This association may occur through *H. pylori*-induced activation of NF-κB and AP-1 proteins which transcriptionally regulate β-catenin expression, responsible for controlling *MYC* expression and consequently cell proliferation [67,80]. On the other hand, *MYC* overexpression was not observed in patients without *H. pylori* infection [79].

**Figure 3:**
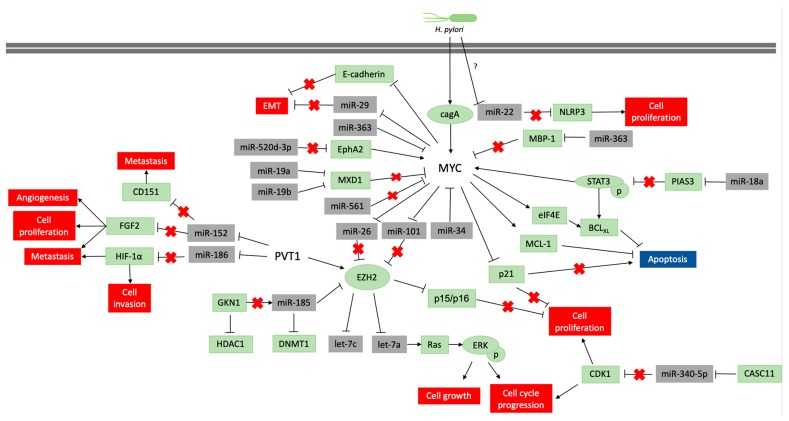
Pathways scheme in which miRNAs regulate MYC and PVT1 expression and vice versa in neoplastic gastric tissue samples and cell lines. The coding genes are shown in green, and the non-coding genes in grey. Lines ending with an arrow indicate activation, whereas T ending lines indicate repression. Lines with a red cross indicate that the interaction is lost due to repression or blocking of a miRNA or protein.

The alteration of the DNA methylation profile is considered to be associated with the *H pylori* inflammatory response, rather than the infection itself [81]. This infection participates in the regulation of *MYC* expression, which is necessary to gastric carcinogenesis occur (Figure 3), but its infection alone is insufficient to the disease establishment. Thus, the identification of molecules and miRNAs associated with *H. pylori* infection in GC can contribute to understand the key cellular and molecular processes at the beginning of carcinogenesis and how environmental factors contribute to GC etiology.

## 3. The Complex Relationship between microRNAs and *MYC* Expression

The deregulation of the expression of several miRNAs can directly or indirectly lead to increased or decreased *MYC*, (Table 2). As described above, *MYC* plays a key role in the normal and tumor development, and the MYC/miRNA network is likely to contribute to the oncogenic functions of MYC. In the present review, we highlight some miRNAs that are altered in GC and are associated with *MYC* activation.

### 3.1. MYC Is Regulated by Epigenetic Modifications

Epigenetic modifications are involved in most cellular biological processes by the regulation of coding and noncoding gene expression. The term epigenetic is defined as heritable modifications in gene expression with no change in the sequence of DNA nucleotides. Notably, *MYC* and miRNAs expressions are regulated by epigenetic mechanisms, such as promoter methylation and histone acetylation. For instance, *miR-212* expression is downregulated by its promoter methylation in GC patient tissues and cell lines. *MYC* mRNA expression is upregulated upon *mir-212* knockdown. Additionally, experiments using wild type and mutant *miR-212* mimics and luciferase activity assays confirmed that *MYC* is a target of *miR-212* [115]. Another example is *miR-33b*, which is downregulated in GC, and its expression can also be inhibited by the hypermethylation of its promoter [116]. Additionally, ectopic expression of *miR-33b* inhibits tumorigenesis in vitro and in vivo by directly targeting MYC, indicating that this miRNA plays a tumor suppressor role in GC (Table 2) [116]. Hayashi et al. showed that the aforementioned *H. pylori cagA* inhibits let-7 expression and enhances the expression of *MYC* DNA methyltransferase 3B (DNMT3B) and Enhancer of Zeste homologue 2 (EZH2). As a result, *let-7* expression was decreased due to methylation of its promoter and increased H3K27 trimethylation [77]. *MiR-448* is another miRNA reported to control *MYC* expression by epigenetic modifications. Hong et al. showed that *miR-448* expression is up-regulated in GC cell lines and patient samples compared to a normal gastric mucous cell line and paired non-tumor tissues. The overall survival and the time to relapse of GC patients with higher *miR-448* expression was reported shorter when compared with the GC patients with low *miR-448*. Consistently, the group of GC tumors with high *miR-448* expression was associated with poor histological differentiation, tumor size and distant metastasis. In vitro and in vivo experiments demonstrated that *miR-448* promotes proliferation by repressing KDM2B (Lysine Demethylase 2B) stimulating glycolysis. Furthermore, KDM2B was shown to be bound to *MYC* promoter inhibiting its expression, demonstrating that KDM2B suppression by *miR-448* is essential for the overexpression of *MYC* expression in GC [117]. A study by Choi et al. revealed that *MiR-185* and GKN1 (Gastrokine 1) expression levels were reduced in gastric mucosal samples, while protein expression levels of *DNMT1, EZH2,* and *MYC* were higher in these samples. Analysis of CpG island methylation showed that *mir-185* and *GKN1* promoters are highly methylated, whereas *DNMT1, EZH2,* and *MYC* promoters are less methylated. Suggesting that *mir-185* may regulate *DNMT1, EZH2,* and *MYC* expression [118]. Collectively, these data suggest that besides epigenetic alterations in its gene, *MYC* expression is also influenced by epigenetic alterations in miRNAs genes.

### 3.2. MicroRNAs Regulate MYC Oncogenic Pathways

MYC is involved in several pathways in gastric tumor cells (Figure 2), and manifold miRNAs may interfere with them, such as *miR-494*, *miR-429*, *miR-520d-3p*, *miR-363, miR-561* and the aforementioned *miR-33b*. In GC, *miR-494* and *miR-429* are downregulated, and negatively correlated with MYC expression. The overexpression of *miR-494* decreased MYC expression, the number of viable cancer cells, tumor burden, and the percentage of proliferative cells [109], and cells transfected with *miR-429* showed downregulation of MYC protein [108]. *miR-520d-3p*, which is also downregulated in GC tissue and cell lines, inhibited the expression of EphA2 (Ephrin Receptor A2), a gene involved in nervous system development. Additionally, cells transfected with mimic *miR-520d-3p* showed lower MYC and EphA2 expression and diminished cell proliferation, invasion, and migration [110,119]. *miR-363* promotes GC progression through MBP-1 inhibition, leading to an upregulation of MYC protein expression [106]. *miR-561* is also downregulated in human GC cell lines and tissues, and its expression was associated with tumor-node-metastasis (pTNM) staging system and suppressed *MYC* expression by directly binding to its 3′-untranslated region [111]. This research group demonstrated that *miR-561* can act as a tumor suppressor miRNA in GC by targeting *MYC* and inhibiting cellular proliferation and invasion. All together these results suggest that these miRNAs can be used as targets for new treatment strategies against GC.

Several studies revealed that the *miR-29* family (*miR-29a-c*) was inversely correlated with *MYC* expression and regulates cell growth and survival by targeting CDK6 (Cell division protein kinase 6), IGF1R (Insulin Like Growth Factor 1 Receptor), TCL1 (T-cell leukemia/lymphoma protein 1A), PI3K (Phosphoinositide 3-kinase) and MCL1 (Induced myeloid leukemia cell differentiation protein) [120,121,122]. However, Gong et al. found that *miR-29* suppresses GC cell proliferation or invasiveness by targeting the cell cycle G1/S transition gene Cyclin D2 (CCND2) or MMP2 (matrix metallopeptidase 2), which encodes for an enzyme that degrades extracellular matrix [122]. The mechanism is unknown yet, but it is well established that MYC directly suppresses *miR-29* [120,123]. As reported by Yan et al., *miR-29b* is recognized as an essential regulator of epithelial-mesenchymal transition (EMT) and is directly involved in cancer metastasis and chemoresistance [124]. Moreover, Saito and colleagues have reported that *miR-29c* is activated by the selective COX2 (cyclooxygenase-2) inhibitors, as Celecoxib, inducing apoptosis, which suggests that *miR-29c* restoring may be a possible treatment for GC [125]. In contrast, Wang et al. found that in GC this miRNA acts as a metastatic suppressors by directly targeting catenin-δ (CTNND1), a gene involved in cell to cell adhesion and signal transduction [126]. These discoveries suggest the differential roles for *miR-29*, not only acting as tumor suppressor miRNA in GC, but also serving as predictors for GC prevention.

MYC can induce *miR-9-3* expression that targets E-cadherin (CDH1), a cellular adhesion protein essential for the cell-cell contact of the gastric epithelium, and promoting tumor cell migration and invasion, leading to EMT in GC [83,127]. The MYC and N-MYC regulation of *miR-9* seems to be one of the main pathways to metastasis [128,129]. In neuroblastoma patients, N-MYC amplification directly correlates with the *miR-9* levels and metastatic spread [128]. On the contrary, *let-7* has target genes [*HMGA2* (High Mobility Group AT-Hook 2), *IMP-1* (IGF2 mRNA-binding protein 1), *LIN28B* (Lin-28 homolog B)*, Ras,* and *MYC*) important for cancer cell stemness [130]. Although *let-7a* expression was high in GC cell lines, it expression diminished progressively during the progression of gastric mucosa cancerization, confirming that the regulation of *let-7a* expression may be used as a novel biomarker and molecular mechanism of drug response to treatment [114]. Enhancer of zeste homolog 2 (EZH2) is a histone methyltransferase involved in the silencing of many genes related to cell proliferation and differentiation. EZH2 in turn enhances H3K27 trimethylation and DNA methylation in *let-7* promoter, and, as a consequence, *let-7a* and *let-7c* expressions are downregulated, resulting in Ras-ERK pathway activation [77]. Additionally, a previous mentioned study showed that GKN1 inhibits EZH2 expression through *miR-185* [118]. GKN1 is considered a tumor suppressor gene as it indirectly inhibits EZH2 and DNMT1, through *miR-185* and directly inhibits the Histone deacetylase 1 (HDAC1). The 8q24.21 *PVT1* oncogene also regulates EZH2 activity. PVT1 recruits EZH2 to occupy the genomic sites of *P15/INK4b* and *P16/INK4b* genes, silencing these gene expressions and enforcing GC cell proliferation [34,131]. The alteration of DNMT1 and HDAC1 expression can lead to demethylation and histone deacetylation, respectively, of several genes important for the maintenance of cell homeostasis, for example, *MYC*, *E-cadherin*, *CDKN1A*, and *CDKN2A* [132,133,134,135].

Other miRNAs such as *miR-135a*, *miR-186*, *miR-494*, *miR-200c*, *miR-374a/b*, *miR-101* and *miR-548* are also targeted by *MYC* gene (Table 2). *miR-101* is downregulated in GC cell lines, and when overexpressed decreases MYC mRNA and protein expression and decreases cell growth, colony formation, and the number of cells in S phase, while increases the number of cells in G1 phase [99]. Similarly, *miR-25* affects the control of cell cycle. Zhang et al. reported *miR-25* upregulation in AGS cells playing an antiapoptotic role as it inhibited FBXW7 and promoted oncogenes, such as *MYC* and cyclin E1 (*CCNE1*), required for G1/S transition [86]. Moreover, the expressions of *CCNE1* and *MYC* are promoted by the upregulation of *miR-25*, suggesting a possible regulatory mechanism of *miR-25* in AGS cells. As mentioned above, cagA enhances *MYC* expression, and MYC regulates EZH2 through *miR-26* and *miR-101* downregulation (Figure 3) [92]. The *H. pylori* infection also suppresses MYC-induced *miR-22* expression in the gastric mucosa, and is associated with an abnormal cell proliferation [91]. *miR-22* is characterized as a key regulator of the self-renewal machinery of the hematopoietic system. This miRNA acts as a proto-oncogenic miRNA via genome-wide deregulation of the epigenetic state through the inhibition of methylcytosine dioxygenase TET2 proteins [136]. On the contrary, *miR-22* is suppressed by *H. pylori* infection, leading to uncontrolled gastric epithelial cell proliferation and overexpression of NLRP3 (NACHT, LRR and PYD domains-containing protein 3), a gene that helps the cell to recognize pathogen-associated molecular patterns [91]. These results indicate that the environment influences the modulation of miRNAs that consequently regulate important pathways of cell proliferation.

The polycistronic miRNA cluster *miR-17-92* encodes six matures miRNAs (*miR-17*; *miR-18a*, *miR-19a*, *miR-20a*, *miR-19b-1*, and *miR-92a-1*), also known as oncomiR-1, and is overexpressed in GC [60]. A mice model that overexpresses the *miR-17-92* cluster developed spontaneous benign tumors in the intestinal tract [88]. Interestingly, MYC functions as a transcriptional factor to *miR-17-92* gene, resulting in the upregulation of this cluster and contributing to *MYC* oncogenic proprieties [60,90,137], and *miR-17* overexpression is associated with MYC in GC tissues [88]. Another example of the importance of this cluster is *miR-29*, which is observed to be downregulated in GC, and its increased tissue expression is associated with higher overall survival rate [93]. *miR-18a*, *miR-19a*, and *miR-19b* are also capable of inducing MYC expression. Ectopic expression of *miR-18a* is capable of retarding gastric tumor growth and angiogenesis through the inhibition of mTOR pathway and is inversely correlated with PIAS3 expression, a STAT3 inhibitor, leading to STAT3p upregulation and induction of MYC expression, with a high predictive value for prognosis of patients with GC [89,138,139]. *miR-19a* and *miR-19b* are also upregulated in GC samples, and directly target MXD1 (MAX Dimerization Protein 1), an important molecule that competes with MYC for MAX (Myc-Associated factor X) binding, impairing the oncogenic MYC-MAX-MXD1 network [140]. Therefore, *miR-19a*/*b* are linked to MYC overexpression through MXD1 inhibition. However, the complete molecular mechanism underlying *miR-17-92* cluster overexpression has not been clearly evaluated in GC [88]. The robust genetic circuitries for the maintenance of cellular dysfunction in GC generate a molecular complex relationship between MYC and miRNAs. This complex relationship implies the control of MYC oncogene pathways leading to tumor development and progression. The studies herein mentioned suggest that miRNAs can not only act directly in one of the main interactions pathways that control MYC expression in GC but also have potential roles as targets for new therapeutic strategies against GC.

Finally, several miRNAs modulate the multidrug resistance of GC, and understanding their role in MYC oncopathway and in the carcinogenesis can elucidate the mechanisms responsible for low survival rate of GC patients. Cao et al. showed that *miR-1284* is downregulated in GC cells, and when it is reintroduced, it enhances the drug-induced apoptosis and impairs the migration and invasion of GC cells [113]. Another example is the down-regulation of *miR-135b*, which was capable of multidrug-resistant protein repression and cell proliferation and induction of cell apoptotic rate of GC cells [141]. Taken together, all the above studies indicate that understanding the complex relationship between MYC and miRNAs is extremely important for gastric carcinogenesis, prognosis and treatment response.

### 3.3. MYC can Promote Angiogenesis through the Regulation of microRNAs

Another interesting point is that m*iR-15a/16-1*, *miR-26a*, *miR-29*, *miR-34a*, and *miR-150* can suppress innumerous survival signaling pathways; however, they are repressed by MYC (Figure 3) [60,84,85]. Moreover, MYC can act as a vascular endothelial growth factor (VEGF) transcriptional factor since it upregulates the expression of proangiogenic factors, promoting angiogenesis and vasculogenesis [142]. Furthermore, in silico data showed that at least eight miRNAs that strictly regulate the VEGF translation are known (*miR-15a*, *miR-16*, *miR-17*, *miR-20a*, *miR-34a*, *miR-93*, *miR-106a*, *miR-106b*), all under MYC control [143]. The VEGF is one of the most important angiogenic growth factors, therefore these results highlight the importance of the deregulation of MYC’s angiogenic properties in the microenvironment during tumors establishment and progression.

## 4. Conclusions

*MYC* and other 8q24.21 genes are associated with GC development and progression. We showed that miRNAs have an influence on the expression of MYC and vice versa. One of the major consequences of *MYC* activation is the extensive reprogramming of the expression pattern of miRNAs in tumor cells, which is closely linked to the modulation of critical pathways associated with cancer etiology, including MYC-MAX-MXD1 and Ras-ERK pathways. Therefore, the studies reviewed revealed that MYC and miRNAs have a very complex interaction in GC that can be affected by external factors, such as bacterial and virus infection. Despite being one of the best-known and studied oncogenes, there is a lack of understanding regarding the application of this information for therapeutic interventions against GC. This application should likely be studied in conjunction with miRNAs expression and action once they appear to be highly correlated. Thus, the investigation of the role of miRNAs helps in the elucidation of underlying mechanisms of gastric carcinogenesis and are potential biomarkers that monitor the alteration of critical genomic driver regions, targeting new treatment strategies against GC.

In fact, besides acting as tumor biomarkers, the miRNAs mentioned in this review may also be used as targets for cancer therapies. Furthermore, there are some examples of miRNA-based therapies currently on clinical trials. A quick search on clinicaltrials.org using the key words “miR” and “cancer” reveals that are 334 studies and many of them are active. For example, the biotechnology company miRagen has been developing therapies using miRNAs: MRG-110, MRG-106 and MRG-201. The first one, MRG-110, uses an LNA-modified antisense oligonucleotides to inhibit *miR-92* and works by increasing the growth of new blood vessels to treat wound healing and heart failure. MRG-106 uses the same technology to silence *miR-155* to treat T-cell lymphoma. MRG-201, in contrast, mimics *miR-29* treat pathological fibrosis, such as keloids and scar tissue formation. Developed by Regulus Therapeutics, RGLS5579 and RG-012 are design to treat patients with glioblastoma multiforme (GBM) and Alport Syndrome, respectively. These drugs target *miR-10b* and *miR-21* inhibiting their function. In addition, there are other less explored approaches, such as using CRISPRi (CRISPR interference) to silence the expression of upregulated miRNAs. In this strategy, a dead-Cas9 fused with transcriptional repressors is targeted to the promoter region of a miRNA, preventing its expression [144,145]. Although there are therapies showing promising results, one of the main challenges in these approaches is to find efficient delivery methods [146]. In this context, a few options are currently been tested, for instance intratumoral injections [147], viral vectors [148], lipid vectors [149], and inorganic nanoparticles [150].

To our knowledge, there are no clinical trials using miRNAs as therapeutic targets for GC to date. Therefore, the characterization of these molecules will be useful in the development of new prognosis, diagnosis, and treatment strategies for patients with GC.

## Figures and Tables

**Table 1:** Genes localized at 8q24.21 region.

Gene	Gene Name	Expression in GC	Reference
Coding genes			
*FAM84B*	Family with sequence similarity 84, member B	?	
*POU5F1B*	POU domain, class 5, transcription Factor 1B	Down	[42]
		Up	[41]
*MYC*	*MYC* proto-oncogene	Up	[14,16,21,22,24,25,26,27,43,44,45]
*GSDMC*	Gasdermin C	?	
*FAM49B*	Family with sequence similarity 49 member B	?	
*ASAP1*	ArfGAP with SH3 domain, ankyrin repeat and PH domain 1	?	
Noncoding genes			
*CASC8*	Cancer susceptibility candidate 8	?	[46]
*CASC11*	Cancer susceptibility candidate 11	Up	[37]
*CASC21*	Cancer susceptibility candidate 21	?	
*CASC19*	Cancer susceptibility candidate 19	?	
*CCAT1*	Colon cancer-associated transcript 1	Up	[38,39]
*CCAT2*	Colon cancer-associated transcript 2	Up	[40]
*LINC00824*	Long intergenic non-protein coding RNA 824	?	
*LINC00977*	Long intergenic non-protein coding RNA 977	?	
*miR-1204*	MicroRNA 1204	?	
*miR-1205*	MicroRNA 1205	Did not differ	[47]
*miR-1206*	MicroRNA 1206	?	
*miR-1207*	MicroRNA 1207	Did not differ	[47]
*miR-1208*	MicroRNA 1208	Did not differ	[47]
*miR-5194*	MicroRNA 5794	?	
*miR-3686*	MicroRNA 3686	?	
*CCDC26*	Coiled-coil domain-containing protein 26	?	
*TMEM75*	Transmembrane protein 75	?	
*PCAT1*	Prostate cancer-associated transcript 1	?	
*PCAT2*	Prostate cancer-associated transcript 2	?	
*PRNCR1*	Prostate cancer associated noncoding RNA 1	?	[48]
*PVT1*	Plasmacytoma variant translocation 1, *MYC* activator	Up	[49]

GC: gastric cancer; Up, upregulated expression in gastric cancer in relation to control; Down, downregulated expression in gastric cancer in relation to nonneoplastic samples; Did not differ, expression in gastric cancer did not differ in relation to nonneoplastic samples; ?: absence of studies on direct relationship between the respective microRNA and MYC in gastric cancer.

**Table 2:** Deregulated miRNAs associated directly or indirectly with MYC expression in gastric cancer.

miRNA		miRNA Expression	MYC Expression	Reference
*miR-9*		Up	Up	[82,83]
*miR-15a/16-1*		Down	Up	[84,85]
*miR-25*		Up	Up	[86,87]
Cluster *miR-17-92*	*miR-17*	Up	Up	[87,88]
	*miR-18a*	Up	Up	[87,89]
	*miR-19a/miR-19b*	Up	Up	[87,90]
	*miR-20*	Up	?	[87]
	*miR-22*	Down	?	[91]
	*miR-26*	Down	Up	[92]
	*miR-29*	Down	?	[93]
	*miR-33b*	Down	Down	[94]
	*miR-34a*	Down	Up	[95,96]
	*miR-92*	Down	?	[97]
*miR-93*		Up	Down	[98]
*miR-101*		Down	Up	[99]
*miR-106a/miR-106b*		Up	?	[87,100]
*miR-150*		Down	Up	[101]
*miR-152*		Down		[102]
*miR-185*		Down	Up	[103]
*miR-186*		Down	?	[104]
*miR-200c*		Down	?	[105]
*miR-212*		Up	Up	[106]
*miR-363*		Down	Up	[106]
*miR-374*		Up	?	[107]
*miR-429*		Down	Up	[108]
*miR-494*		Down	Up	[109]
*miR-520d-3p*		Down	Up	[110]
*miR-561*		Down	Up	[111]
*miR-935*		Up	Up	[112]
*miR-1284*		Down	?	[113]
*let-7a*		Up	Up	[114]

Up, upregulated expression in gastric cancer in relation to control; Down, downregulated expression in gastric cancer in relation to control; ?: absence of studies on direct relationship between the respective microRNA and MYC in gastric cancer.

## Abbreviations

GC	Gastric cancer
miRNA	microRNA
RISC	RNA-induced silencing complex
H. pylori	Helicobacter pylori
IARC	International Agency for Research on Cancer
EMT	Epithelial-mesenchymal matrix
GBM	Glioblastoma multiforme
CRISPRi	CRISPR interference
